# Clinical Significance of Telomerase Reverse-Transcriptase Promoter Mutations in Hepatocellular Carcinoma

**DOI:** 10.3390/cancers13153771

**Published:** 2021-07-27

**Authors:** Francesca Pezzuto, Francesco Izzo, Pasquale De Luca, Elio Biffali, Luigi Buonaguro, Fabiana Tatangelo, Franco Maria Buonaguro, Maria Lina Tornesello

**Affiliations:** 1 Molecular Biology and Viral Oncology Unit, Istituto Nazionale Tumori IRCCS Fondazione G. Pascale, 80131 Napoli, Italy; francesca.pezzuto@istitutotumori.na.it (F.P.); f.buonaguro@istitutotumori.na.it (F.M.B.); 2 Hepatobiliary Surgery Unit, Istituto Nazionale Tumori IRCCS Fondazione Pascale, 80131 Napoli, Italy; f.izzo@istitutotumori.na.it; 3 Research Infrastructures for Marine Biological Resources, Stazione Zoologica Anton Dohrn, 80122 Napoli, Italy; pasquale.deluca@szn.it (P.D.L.); elio.biffali@szn.it (E.B.); 4 Cancer Immunoregulation Unit, Istituto Nazionale Tumori IRCCS Fondazione G. Pascale, 80131 Napoli, Italy; l.buonaguro@istitutotumori.na.it; 5 Department of Pathology, Istituto Nazionale Tumori IRCCS Fondazione Pascale, 80131 Napoli, Italy; f.tatangelo@istitutotumori.na.it

**Keywords:** TERT promoter, TERTp, hepatocellular carcinoma, HCC, droplet digital PCR, ddPCR, hepatitis B virus, HBV, hepatitis C virus, HCV, cirrhosis, mutation

## Abstract

**Simple Summary:**

Activating mutations in the promoter region of TERT (TERTp) gene are frequently observed in low- and high-grade dysplastic nodules and defined as early events in hepatocellular carcinoma development. This study shows that the nucleotide change G>A at position −124 in the TERTp region is very common in hepatocellular carcinoma. The concordance rate between droplet digital PCR (ddPCR) (63.6%) and Sanger sequencing (52.1%) detection methods is good (83.5%). HCC patients carrying the TERTp mutation had lower levels of the tumour biomarker Ca19-9 but showed reduced survival. The presence of TERTp mutations may represent a prognostic signature in liver cancer.

**Abstract:**

Telomerase reactivation during hepatocarcinogenesis is recurrently caused by two point mutations occurring most frequently at the nucleotide −124 (95%) and occasionally at the nucleotide −146 (<5%) upstream of the TERT translational start site in hepatocellular carcinoma (HCC). In this study, we designed a droplet digital PCR (ddPCR) assay to detect TERT promoter (TERTp) nucleotide change G>A at position −124 and to quantify the mutant allele frequency (MAF) in 121 primary liver cancers, including 114 HCC along with 23 autologous cirrhotic tissues, five cholangiocarcinoma (CC), and two hepato-cholangiocarcinoma (HCC-CC). All cases were evaluated for tumour markers such as α-fetoprotein (AFP), carbohydrate antigen 19-9 (CA19-9), and carcinoembryonic antigen (CEA). We compared the sensitivity of ddPCR and Sanger sequencing and investigated the prognostic relevance of TERTp mutations. The TERTp G>A transition was identified in 63.6% and 52.1% of HCC samples by ddPCR and Sanger sequencing, respectively. One out of 23 (4.3%) peri-tumour tissues tested positive only by ddPCR. One out of five CC (20%) and none of the HCC-CC were found concordantly mutated by the two methods. The TERTp MAF ranged from 2% to 66%, and the large majority (85.5%) of mutated samples showed a value above 20%. A statistically significant correlation was found between TERTp mutation and tumour size (*p* = 0.048), while an inverse correlation was observed with CA19-9 levels (*p* = 0.0105). Moreover, HCC patients with TERTp −124A had reduced survival. In conclusion, the single nucleotide variation G>A at position −124 in TERTp, detected either by ddPCR or by Sanger sequencing, showed a remarkable high frequency in HCC. Such mutation is associated with lower levels of CA19-9 and reduced survival in HCC patients suggesting that the TERTp status may represent a distinct signature of liver cancer subgroups.

## 1. Introduction

Primary liver cancer with a global burden of 905,677 new cases and 830,180 deaths in 2020 is the second and the sixth leading cause of cancer mortality in men and in women, respectively [1]. Major risk factors for liver cancer development are chronic infections with hepatitis B (HBV) and C (HCV) viruses, causing approximately 80% of all cases worldwide, and non-viral factors, including heavy alcohol consumption, non-alcoholic fatty liver disease (NAFLD), and dietary exposure to aflatoxin B1 [2,3,4]. Hepatocellular carcinoma (HCC) represents the most common primary liver malignancy with approximately 90% of all cases in the world [5]. Cholangiocarcinoma (CC), developing from the biliary epithelium, and combined hepatocellular-cholangiocarcinoma (HCC-CC) are relatively rare accounting for approximately 10% and 4%, respectively [6].

Liver cancer diagnosis remains a challenge, since imaging techniques, such as computed tomography and dynamic contrast enhanced magnetic resonance, as well as dosage of serum biomarkers, such as α-fetoprotein (AFP), carbohydrate antigen 19-9 (CA19-9), and carcinoembryonic antigen (CEA), have low sensitivity and specificity for the diagnosis of small neoplastic nodules [7]. Surgical therapeutic approaches, including tumour resection, liver transplantation, local ablation, or trans-arterial chemoembolization and intra-arterial infusion are effective only in patients with early or intermediate stage HCC [8]. However, the large majority of tumours are diagnosed at late stages and tyrosine kinase inhibitors, such as sorafenib and lenvatinib, as first-line therapies and cabozantinib and regorafenib as second-line treatments have shown to increase the median overall survival to above eighth months [9,10,11]. The combination of the anti-PDL1 antibody atezolizumab and the anti-VEGF antibody bevacizumab with kinase inhibitors has more than doubled the life expectancy in treated HCC patients [12]. In addition, the immune-checkpoint inhibitor nivolumab administered as single agent has shown to provide significant benefit in up to 20% of treated patients, although no specific biomarkers were identified to select responder patients [13]. Therefore, the study of molecular features of liver cancer is fundamental for the identification of signatures predictive of patient outcome and for the selection of actionable targets for cancer therapy.

Comprehensive genomic studies recognised the high molecular heterogeneity of HCC resulting from the complex interplay between viral and non-viral risk factors along with host susceptibility, such as the genetic polymorphisms in the immune response genes [14,15,16]. Mutations in oncosuppressors and oncogenes have shown to be relatively frequent in HCC and to affect more than 10 signalling pathways defined as major players of hepatocarcinogenesis [17]. Indeed, inactivation of the TP53-RB pathway observed in approximately 30% of HBV-related HCC is a common event due to frequent mutations in TP53 gene as well as in ATM and RPS6KA3, encoding p53-activating kinases [17,18,19]. The WNT pathway has been found commonly deregulated in HCV-related HCC due to activating mutations in CTNNB1 gene and inactivating mutations in WNT regulators, including AXIN1 and APC genes [17,20].

Cancer-specific nucleotide variations in the promoter region of TERT gene, first identified by Horn et al. (2013) and Huang et al. (2013), were shown to occur more frequently than any other observed somatic mutation in several cancer types including HCC [21,22,23]. Telomerase expression and telomeres elongation play pivotal roles either in physiological hepatic regeneration or in liver cancer development [24,25]. In vivo studies showed that only a small subset of hepatocytes expressing physiologically high levels of telomerase are able to repopulate the liver tissue in response to hepatic damages and their cell progeny to return at repressed condition of TERT expression [26]. TERTp mutations, mainly the nucleotide change G>A at position −124 in TERTp, were shown to irreversibly activate TERT gene and to give rise to hepatocyte clones with permanent telomerase over-expression and promotion of uncontrolled growth [27].

The TERTp mutation −124A represents approximately 95% of all TERTp variations in HCV-related HCC and in metabolic-related HCC [28]. Moreover, TERTp nucleotide changes are considered “trunk mutations” in liver carcinogenesis being frequently identified in hepatic precancerous lesions and at increased frequency in HCC [29,30].

End-point PCR and Sanger sequencing are defined as gold standard for the detection of mutations in target DNA regions, although this method is not quantitative and has low sensitivity for the identification of rare mutants [31]. The digital polymerase chain reaction, based on the principle of limiting dilution and Poisson statistics, is a third-generation PCR technology for absolute quantification of DNA target sequences [32]. In particular, the droplet digital PCR (ddPCR) consists of the partitioning of the sample into up to 20,000 water-in-oil discrete “droplets”, each containing zero, one, two, or more copies of target nucleic acids, in the independent amplification and counting of nucleic acid molecules encapsulated in the partitions [33]. The ddPCR is applicable to measure mutant allele fractions and to classify cancer driver mutations as “trunk” or “branch” events as well as being suitable for the detection of rare DNA variants appearing in the early phases of malignant cell transformation [34,35].

In this study, we developed a probe-based droplet digital PCR (ddPCR) assay to detect the mutation TERTp −124A. Then, we compared the results with the TERTp mutation profile determined by Sanger sequencing analysis in liver cancer samples, including tumour and peri-tumour paired biopsies. Moreover, we evaluated the TERTp mutation allele frequency (MAF) and the relationship between TERTp mutational status with clinical–pathological parameters of liver cancer patients included in this study.

## 2. Materials and Methods

### 2.1. Patients and Tissue Samples

The study comprised 121 consecutive patients in BCLC stage A or stage B who were treated by surgical liver resection according to Milan criteria at the Hepatobiliary Unit of the Istituto Nazionale Tumori “Fondazione G. Pascale”. Tumour biopsies were obtained from patients diagnosed with HCV-related HCC (*n* = 99), HBV-related HCC (*n* = 10), and non-virus related HCC (*n* = 5 including one alcohol-related case, one non-alcoholic steatosis hepatitis (NASH) HCC, one steatosis HCC, and two cases of unknown aetiology), HCV-related CC (*n* = 5), and HCC-CC (*n* = 2). Autologous peri-tumour cirrhotic tissues were obtained from 23 cases of HCV-related HCC. Control liver tissues were obtained from 11 healthy patients. Ninety out of 121 liver cancer cases and the 11 controls have been previously analysed for TERTp and CTNNB1 mutations [36].

Patients were classified according to their Child–Pugh score into A (*n* = 98) and B (*n* = 23). At the time of tumour resection, each liver biopsy was divided by the pathologist in two sections: the first section was sub-fragmented and stored in RNAlater^®^ solution (Ambion, Inc., Austin, TX, USA) at −80 °C, while the second was subjected to histopathologic examination. Liver cancers were classified into three groups according to the histological grade in well differentiated (G1, *n* = 1), moderately differentiated (G2, *n* = 109), and poorly differentiated (G3, *n* = 3; G4 *n* = 1) according to the criteria of Edmondson and Steiner [37]. Patient information and biomarker test results, including AFP, CA19-9, CEA, ALT, AST, and GGT, were collected retrospectively. Preoperative blood testing for tumour biomarkers was carried out with regulatory agency-approved and commercially available kits according to the manufacturers’ instructions. The upper limits of tumour biomarkers standard reference values were AFP ≤ 20 ng/L, CEA ≤ 3 ng/L, and CA19-9 ≤ 37 U/mL.

The study is in accordance with the principles of the Declaration of Helsinki, and it was approved by the Institutional Scientific Board and by the Ethical Committee of the Istituto Nazionale Tumori “Fond Pascale” (N. 421/13). All patients provided written informed consent to participate to the study.

### 2.2. DNA Extraction

DNA was extracted from frozen tissue samples by digestion with proteinase K (150 μg/mL) in 500 μL of lysis buffer (10 mM Tris-HCL, pH 7.6, 5 mM EDTA, 150 mM NaCl, 1% SDS) at 37 °C, overnight. After digestion, DNA was purified with phenol-chloroform-isoamyl alcohol (25:24:1) extraction and ethanol precipitation in 0.3 M sodium acetate (pH 4.6). The purified DNA samples were analysed by Nanodrop 2000c spectrophotometer (Thermo Fisher Scientific, Waltham, MA, USA) to assess the quality and quantity of nucleic acids. The ratio of absorbance at 260 nm and 280 nm was equal or above 1.8 for all DNA samples.

### 2.3. TERTp Mutation Analysis by End-Point PCR and Sanger Sequencing

TERTp region was amplified using primer pairs and protocols described previously [30]. Briefly, the oligo primers hTERT-F (5′-ACGAACGTGGCCAGCGGCAG-3′) and hTERT-R (5′-CTGGCGTCCCTGCACCCTGG-3′) were used to amplify a 474 bp fragment comprising the nucleotides −124 and −146 bp before TERT ATG start site. PCR negative samples were further amplified with oligonucleotides hTERT_short_F (5′-CAGCGCTGCCTGAAACTC-3′) and hTERT_short_R (5′-GTCCTGCCCCTTCACCTT-3′) amplifying a 163 bp fragment within TERTp region. PCR reactions were performed in 50 µL reaction mixture containing 100–300 ng of genomic DNA, 10 pmol of each primer, 1.25 Unit of Hot Master Taq DNA Polymerase (5 Prime GmbH, Hamburg, Germany), and 25 µL of PreMix J (Master Amp PCR, Epicentre, Singapore). DNA was amplified in Sure Cycler 8800 thermal cycler (Agilent Technologies, Santa Clara, CA, USA) with an initial denaturation at 94 °C for 3 min, followed by 45 amplification cycles of annealing at 65 °C for 30 s, elongation at 72 °C for 1 min, denaturation at 94 °C for 30 s, and 10 min final elongation at 72 °C. All the PCR amplification products were subjected to automated bi-directional direct sequencing analysis (Eurofins Genomics GmbH, Ebersberg, Germany).

### 2.4. TERTp Mutation Analysis by ddPCR

The “Minimum Information for Publication of Quantitative Digital PCR Experiments for 2020” (dMIQE2020) checklist is provided in Supplementary Table S1 [38].

The analysis of the mutation TERTp −124A was performed with a probe-based assay containing TERT forward 5′-CGCGGAAAGGAAGGG-3′ and TERT reverse 5′ACCCCTCCCGGGTCC primers as well as mutant and wildtype FAM or HEX labelled probes (TERT −124A 5′-CCCGGAAGGGGCTGGG-3′; TERT −124G (wildtype) 5′-CCCGGAGGGGGCTGG-3′). The ddPCR reactions were carried out in 20 μL volumes consisting of 10 μL 2x ddPCR Super Mix for probes (no dUTP) (Bio-Rad Laboratories, Hercules, CA, USA), 2.5 μL (100 ng) DNA template, 1 μL (900 nM/250 nM/250 nM) primers/probe −124A/probe −124G mix (Bio-Rad Laboratories, Hercules, CA, USA), 2 μL of 5 M Betaine solution (Sigma Aldrich, St. Louis, MO, USA) and 3.5 μL of deionized distilled water. Each 20 μL reaction volume was carefully loaded into the well of a droplet generator cartridge (Bio-Rad Laboratories, Hercules, CA, USA), and 70 μL droplet generation oil (Bio-Rad Laboratories, Hercules, CA, USA) were subsequently loaded to generate droplets. The cartridge was covered with Droplet Generator Gasket, inserted into QX200 Droplet Generator (Bio-Rad Laboratories, Hercules, CA, USA) to generate up to 20,000 droplets from each sample and transferred into a 96-well PCR plate. The amplification reaction consisted of 10 min denaturation at 95 °C, followed by 40 cycles of 1 min annealing/extension at 55 °C and 30 s denaturation at 94 °C, ending with a final cycle of 10 min denaturation at 98 °C and holding at 4 °C. The rate of temperature rise was set at 2 °C/s. After the amplification, the fluorescent signals in the 96-well plate were acquired by the QX200 Droplet Reader (Bio-Rad Laboratories, Hercules, CA, USA) and signals analysed using the QuantaSoft software version 1.7.

The specificity of the TERTp mutation assay was evaluated by testing mutant and wildtype templates validated by orthogonal methods (i.e., Sanger sequencing). Sensitivity and limit of detection (LOD) was calculated by testing serial dilution of mutant DNA into wildtype DNA (50%; 25%; 12.5%; 6.25%; 3.12%; 1.56%; 0.78%; 0.39%; 0.19%; 0.095%) (Figure S1) and linear regression analysis. Each dilution was run in three replicates and analysed as a metawell. The limit of blank (LOB) was calculated by determining the false-positive mean and the relative standard deviation of the TERTp −124A assay in 11 replicates of genomic DNA (100 ng) extracted from liver tissues from healthy individuals (Figure S3). The thresholds for positives TERTp −124A and TERTp wildtype were set to 2000 for each reaction. Then, the mutant allele concentration (copies/20 μL, CMUT) and wildtype allele concentration (copies/20 μL, CWT) were used to calculate the mutant allele frequency (MAF) as MAF = CMUT/(CMUT + CWT).

### 2.5. Statistical Analysis

Statistical analysis was performed using EpiInfo Version 6 and Graphpad Prism 6 software. HCC patients were stratified by mutational status, sex, age, tumour grade, and hepatitis virus infection. Comparison between groups was performed using Mantel Haenszel corrected χ^2^ test. Results obtained by ddPCR analysis were compared to results obtained by end-point PCR and subsequent Sanger sequencing by employing Cohen’s Kappa test. Concordance was considered excellent when comprised between 0.81 and 1, good when comprised between 0.61 and 0.81 and moderate when comprised between 0.41 and 0.60. Data on survival were available for 53 HCC patients. Survival rates were estimated using the log-rank test (Mantel–Cox). Overall survival was defined as the period between the time of surgery and death. Living patients were censored with the date of their last follow-up. *p*-values less than 0.05 were considered statistically significant.

## 3. Results

We developed an in-house-designed assay to identify and quantify the nucleotide variation G>A at position −124 upstream the TERT gene ATG start site by ddPCR. The amplification protocol was optimized by testing a range of annealing temperatures (50 °C–60 °C) and betaine concentrations (0.125–0.5 M).

**Figure 1:**
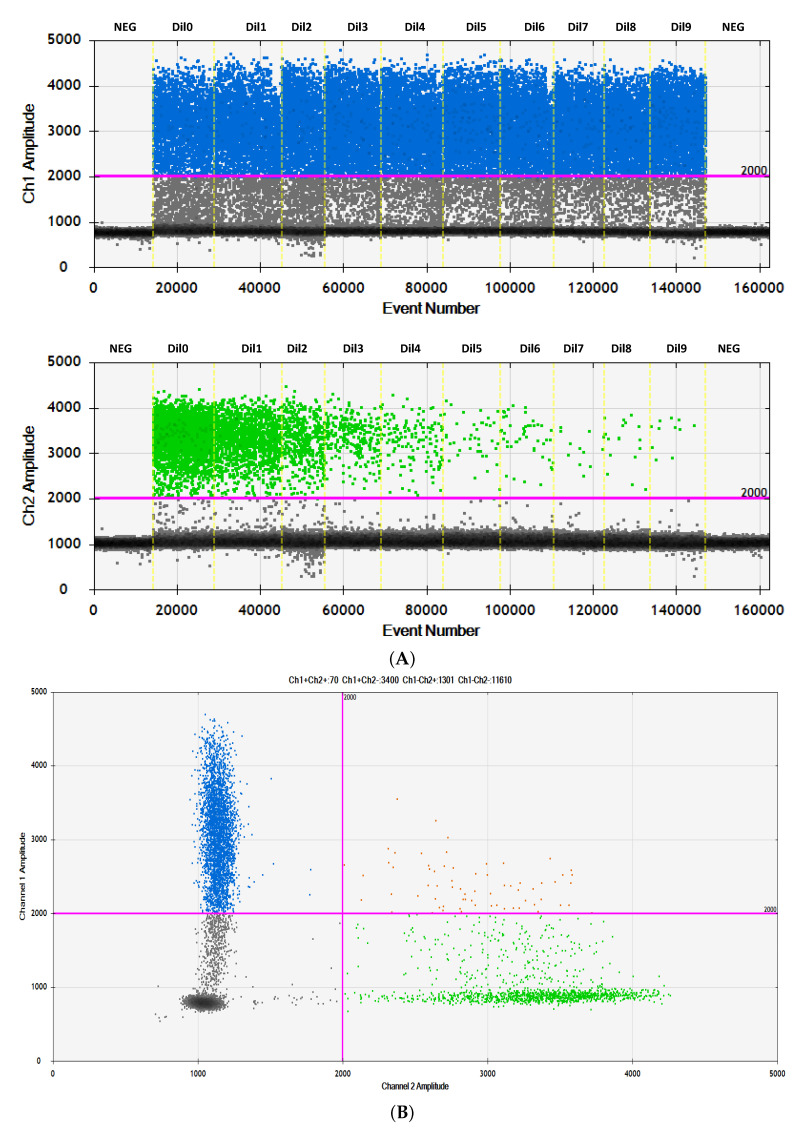
(**A**) 1D plot showing ddPCR amplification of serial dilutions of the HCC DNA sample 433 carrying the heterozygous mutation −124A in TERTp as determined by Sanger sequencing in the background of TERTp wildtype DNA (NEG, No DNA; Dil0, 50%; Dil1, 25%; Dil2, 12.5%; Dil3, 6.25%; Dil4, 3.12%; Dil5, 1.56%; Dil6, 0.78%; Dil7, 0.39%; Dil8, 0.19%; Dil9, 0.095%). (**B**) 2D amplitude plots of the raw data are shown for ddPCR 25% −124A/WT. Green: HEX probe for TERTp −124A; blue: FAM probe for TERTp WT; orange: double positive droplets containing both TERTp −124A and TERTp wildtype alleles (−124G); grey: droplets negative for template DNA. The pink line is a manually set threshold.

The sensitivity of the assay was measured by performing serial dilutions of TERTp mutated genomic DNA (sample 433) into wildtype genomic DNA, extracted from liver control tissue (sample 70) that allowed us to set the limit of detection (LOD) to 0.2% MAF by linear regression analysis (Figure 1; Figure S1).

The analytical performance of the ddPCR TERTp −124G/A assay was evaluated by testing the HCC DNA samples previous analysed by orthogonal methods such as Sanger sequencing (Table 1). The concordance rate for TERTp −124A detection between the two methods was 83.5% (Table 2). The Cohen’s kappa coefficient was 0.666 (95% CI 0.536 to 0.796) suggesting a good agreement. Among the 20 discordant cases, three mutant samples were negative by ddPCR and positive by Sanger sequencing, 17 cases were positive only by ddPCR.

The study included 121 liver cancer patients comprising 114 HCC, five CC, and two HCC-CC cases (Table 3). The majority of patients were males (75.4%) with a mean age of 67.5 years (SD ± 7.3). The HCV infection was the main cause of HCC (86.8%) and CC/HCC-CC (57.1%).

**Figure 2:**
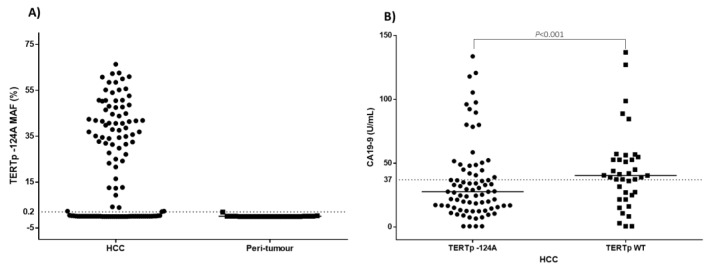
(**A**) Mutation allele frequency (MAF, %) of TERTp −124A in HCC and peri-tumour tissues. Black dashed line indicates the lower limit of detection of TERTp −124A assay. (**B**) CA19-9 levels in HCC patients with TERTp wildtype (WT) and TERTp −124A. The black dashed line indicates the upper limit of CA19-9 reference intervals. The black line indicates the mean value in each group.

Overall, the TERTp −124A mutation was detected in 79 (69.3%) HCC, one out of five (20%) CC and none of the two HCC-CC cases by using both techniques. The mutant allele frequency (MAF) ranged from 0.20% to 66% (Figure 2). The majority of mutated cases showed a MAF higher than 20% supporting the datum that TERTp mutation is a “trunk event” in hepatocarcinogenesis. Eighteen cases with TERT −124A MAF above 50% were characterized by an advanced stage HCC. The autologous peri-tumour tissues were found mutated only in one case (MAF = 1.9%) out of 23 samples and in none of the 11 control tissues.

HCV-related cases were more frequently mutated (72.7%) than HBV-related cases (60%). Among non-virus-related HCC, only one out of five (20%) presented the −124A substitution.

We observed no significant association between TERTp mutation and GGT, ALT, and AST as well as AFP and CEA blood levels; however, a higher frequency of TERTp −124A was observed among patients with lower levels of CA19-9 (*p* = 0.0105). On the other hand, the TERTp mutation frequency was statistically significant correlated with HCC tumour size (*p* = 0.0428).

**Figure 3:**
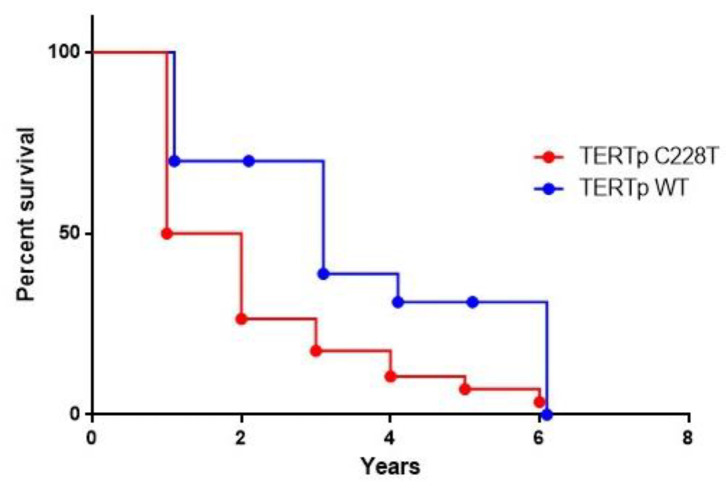
Survival percentages were compared using the log-rank test (Mantel–Cox). HCC cases with TERTp −124A mutation had a reduced survival (median survival = 18 months) compared to not-mutated cases (median survival = 36 months). (*p* = 0.0159; Mantel–Haenszel hazard ratio (HR) 2.397; 95% CI 1.18–4.88.

Data on survival were available for 53 HCC patients. The Mantel–Cox log rank test showed that HCC cases with TERTp −124A had a reduced survival (median survival = 18 months) compared with patients with non-mutated tumours (median survival = 36 months), suggesting a correlation between TERTp mutation burden and poor prognosis (*p* = 0.0159; Mantel–Haenszel hazard ratio (HR) 2.397; 95% CI 1.18–4.88 (Figure 3).

## 4. Discussion

We have designed a ddPCR TERTp −124A assay and analysed liver cancer cases previously evaluated by end-point PCR and Sanger sequencing [36]. There was a good concordance between the two methods with ddPCR being more sensitive (63.6%) compared to Sanger sequencing (52.1%). The mutant allele frequency in each sample was generally higher than 20% indicating that TERTp nucleotide changes represent “trunk mutations” with cancer driver functions in HCC. Indeed, TERTp mutations have been recognized as early events in liver cancer development, since they are identified at lower frequency in pre-neoplastic nodules arising in the cirrhotic liver and at an increasingly higher frequency in HCC progression [29].

Telomerases elongation and telomerase activity are essential for cancer development [39]. In liver cancer, the expression of the TERT gene is activated by different mechanisms including focal amplification, rearrangements, and mutations in the TERT promoter region. In HBV-related HCC, the telomerase is frequently activated by the insertional mutagenesis of the HBV genome; therefore, the rate of TERTp mutations is generally lower than that observed in HCV-related and non-viral HCCs [40]. Accordingly, a systematic meta-analysis, evaluating the distribution of TERTp mutations in 1939 primary HCC from four continents, demonstrated that such nucleotide changes are very common in the HCC of various aetiology, with mutation rates higher in Europe (56.6%) than Asia (42.5%), the latter cases being predominantly related to HBV infection and TERT gene altered by the virus integration in the human genome [41].

TERTp mutations and high telomerase expression have been associated with poor prognosis in several tumours, such as gliomas, thyroid carcinoma, non-small cell lung cancer, colorectal cancer, and soft tissue sarcomas [42]. In our study, HCC patients with TERTp −124A had a reduced survival (median survival = 18 months) compared with patients with non-mutated tumours (median survival = 36 months), suggesting a correlation between TERTp mutation and poor prognosis (*p* = 0.0159). However, the role of telomerase activity in the aggressiveness of HCC is controversial. Recently, Ningarhari et al. (2021) investigated the telomere length in the tumour and non-tumour liver tissues of more than 1500 patients and its relationship with TERT genetic alterations and expression as well as with HCC molecular features and clinical outcome [43]. They observed that TERT mRNA is elevated in 89.1% of HCCs and marginally associated with telomere length. In addition, the frequency of somatic TERTp mutations was high among the HCCs of the non-proliferative subclass, which are less aggressive tumours developing in livers characterized by short telomeres, well-moderate histological differentiation, low levels of AFP, infrequent vascular invasion, and chromosomal stability. Lower rates of TERTp mutations, instead, were observed in the proliferative HCC class, arising in livers characterized by longer telomeres, categorized as more aggressive tumours with poor histological differentiation, high vascular invasion, and increased levels of AFP [43].

In our study, no significant association was found between TERTp mutation frequency and levels of tumour biomarkers AFP and CEA. A statistically negative correlation, instead, was observed between TERTp −124A and CA19-9 levels. The tumour-associated antigen CA19-9 is a glycoprotein mainly produced by the pancreatic duct, gastrointestinal tract epithelium, and biliary system [44]. In HCC patients, the elevated levels of CA19-9 before surgery have been associated with worse survival in patients who were resected or underwent liver transplantation. Hsu et al. showed that serum levels of CA19-9 above 100 U/mL was an independent risk factors for prediction of poor overall survival of HCC patients and that it was associated with a 2.6-fold increased mortality. Therefore, TERTp wildtype HCC in patients with high levels of circulating CA19-9 may be useful to classify a subgroup of aggressive liver tumours [45].

A significant co-occurrence of TERTp mutations and CTNNB1 gene mutations in HCV-related HCC has been reported by several studies. However, the relevance of this association in terms of biological mechanisms and response to therapies has not yet been investigated. Nevertheless, TERTp mutations and BRAF V600E variation have been shown to have a synergistic oncogenicity through the axis BRAF V600E/MAP kinase pathway/FOS/GABP causing activation of mutant TERTp [46]. Importantly, the presence of both mutations has been shown to induce a robust apoptosis in thyroid, melanoma, and colon cancer cells and to abolish near completely their growth in vivo following combined treatment with dabrafenib and trametinib [47]. The drugs had little effect on tumours harbouring only BRAF V600E. Further studies are needed to reveal possible interplay between mutated TERTp and genetic alterations in cancer driver genes that could activate new actionable pathways for targeted therapies in HCC.

There are some limitations of the present study. First, we used tumour fragments to obtain DNA, which may have misclassified some sub-clonal TERTp mutations. Indeed, three samples found mutated by Sanger sequencing were negative by ddPCR. The new sampling protocols based on the homogenization of tumour materials and demolition of clonal structures are the best approach to overcome the tumour heterogeneity and to obtain a full mutation profile by current sequencing protocols [48]. Second, many patients have not received follow-up after surgical treatment; therefore, the available data are insufficient to fully evaluate the correlation between TERTp −124A and overall survival or progression free survival.

## 5. Conclusions

We confirmed by ddPCR analysis that TERTp −124A has a very high frequency in liver cancers of various aetiology, particularly in HCV-related cases. Moreover, TERTp mutation may be employed as a predictive biomarker due to the early occurrence of this mutation in hepatic carcinogenesis and the correlation with a reduced survival. Further standardization and multicentre validation are necessary to implement such strategy in the routine clinical practice of liver cancer management.

## Figures and Tables

**Table 1:** Detection of TERTp −124A mutation by ddPCR and Sanger sequencing in DNA samples extracted from liver cancer biopsies.

Sample ID	Sanger TERTp	ddPCR TERTp	MAF (%)	Number of Alleles Screened	Sample ID	Sanger TERTp	ddPCR TERTp	MAF (%)	Number of Alleles Screened
57	WT	WT	-	3084	307	−124A	−124A	41.71	4057
60	WT	WT	-	1934	314	WT	WT	-	4620
66	−124A	−124A	47.48	7736	315	−124A	−124A	41.42	10285
78	WT	WT	-	8560	324	−124A	−124A	43.76	4991
86	−124A	−124A	39.82	1871	334	WT	−124A	24.98	8406
88	WT	−124A	0.36	4876	338	−124A	WT	-	5279
117	−124A	−124A	54.05	8145	339	−124A	−124A	38.62	6007
126	WT	−124A	0.23	2645	340	−124A	−124A	27.70	6112
128	WT	−124A	2.32	1813	342	−124A	−124A	35.67	7580
129	−124A	−124A	44.57	3812	353	WT	WT	-	4207
132	−124A	−124A	12.45	1935	354	−124A	−124A	47.74	5754
134	WT	−124A	2.26	5493	355	WT	WT	-	6078
137	WT	−124A	0.35	1733	361	WT	−124A	52.61	6441
138	WT	WT	-	263	365	−124A	−124A	29.85	4798
144	WT	−124A	4.20	940	366	−124A	−124A	31.47	4894
146	−124A	−124A	12.70	4024	369	WT	WT	-	5635
152	−124A	WT	-	1220	370	−124A	−124A	37.65	8123
153	WT	WT	-	5077	372	−124A	−124A	31.92	4533
157	WT	−124A	0.32	2479	373	−124A	−124A	58.54	10,925
158	WT	−124A	12.28	477	374	−124A	−124A	35.13	4535
164	WT	−124A	9.26	1614	375	−124A	−124A	60.81	5586
167	−124A	−124A	44.94	6662	377	WT	WT	-	4255
169	WT	−124A	0.54	2508	382	−124A	−124A	50.30	6402
170	WT	WT	-	1857	386	−124A	−124A	34.90	4433
172	−124A	−124A	16.43	2258	387	WT	WT	-	6664
174	WT	WT	-	773	391	WT	WT	-	3067
175	WT	−124A	36.88	1214	392	−124A	−124A	36.87	3699
177	WT	WT	-	2357	393	−124A	−124A	50.49	4710
179	−124A	−124A	21.58	5584	394	WT	WT	-	7026
180	−124A	−124A	42.45	940	396	−124A	−124A	62.57	4133
181	−124A	−124A	41.86	6366	398	WT	WT	-	5286
186	−124A	−124A	0.25	3257	399	−124A	−124A	35.37	4241
187	−124A	−124A	40.60	298	402	−124A	−124A	50.72	4237
189	−124A	−124A	60.02	4650	403	−124A	−124A	48.65	10,681
190	−124A	−124A	62.28	920	404	−124A	−124A	52.82	4806
193	−124A	−124A	34.05	1536	406	WT	WT	-	3916
195	−124A	−124A	32.68	4932	407	WT	−124A	0.45	5839
197	−124A	−124A	55.21	3735	408	−124A	−124A	31.41	7501
199	−124A	−124A	55.70	4501	409	−124A	−124A	41.49	3155
203	WT	WT	-	1641	411	WT	WT	-	4043
206	WT	WT	-	3139	412	WT	WT	-	6960
208	−124A	−124A	50.61	4545	414	−124A	WT	-	5194
211	−124A	−124A	41.87	8866	415	WT	WT	-	3871
212	WT	WT	-	2403	416	WT	WT	-	7303
221	−124A	−124A	58.54	6917	417	WT	WT	-	4082
226	WT	WT	-	5609	418	−124A	−124A	55.15	7318
229	WT	WT	-	5934	421	WT	WT	-	5523
233	WT	WT	-	3502	422	−124A	−124A	37.95	5658
235	−124A	−124A	27.10	5022	423	−124A	−124A	66.37	11,773
236	WT	WT	-	5060	424	WT	WT	-	6444
241	WT	WT	-	4670	425	−124A	−124A	48.11	6117
242	WT	WT	-	1217	426	WT	−124A	40.73	5949
243	−124A	−124A	34.54	4100	427	−124A	−124A	40.57	9056
245	WT	WT	-	4708	428	WT	WT	-	6447
247	−124A	−124A	16.22	2226	429	WT	−124A	2.01	5240
248	−124A	−124A	0.41	2089	430	WT	WT	-	8393
259	−124A	−124A	3.37	4388	431	WT	WT	-	6915
273	−124A	−124A	47.21	5484	432	WT	WT	-	15,202
282	−124A	−124A	32.43	3170	433	−124A	−124A	50.68	7675
287	WT	−124A	2.95	4738	434	WT	WT	-	5818
297	WT	WT	-	3814					

**Table 2:** Concordance rate between Sanger sequencing and ddPCR assay.

Sanger vs. ddPCR	Sanger Sequencing			Performance	
ddPCR	TERTp −124A	TERTp wildtype	Total	Sensitivity	95.2%
TERTp −124A	60	17	77	Specificity	70.7%
TERTp wildtype	3	41	44	Concordance	83.5%
Total	63	58	121		

**Table 3:** Correlation between TERTp −124A mutation status and clinic-pathological variables in HCC patients.

Characteristics	HCC	TERTp WT (*n* = 35)	TERTp −124A (*n* = 79)	*p* Value *
	(*n* = 114)			
Age (years), *n* (%)				0.116
≤65 years	37 (32.5)	15 (42.9)	22 (27.8)	
>65 years	77 (67.5)	20 (57.1)	57 (72.2)	
Gender, *n* (%)				0.259
Male	86 (75.4)	24 (68.6)	62 (78.5)	
Female	28 (24.6)	11 (31.4)	17 (21.5)	
Etiology, *n* (%)				0.387
HBV+	10 (8.8)	4 (11.4)	6 (7.6)	
HCV+	99 (86.8)	27 (77.1)	72 (91.1)	
No virus	5 (4.4)	4 (11.4)	1 (1.3)	
AFP (ng/mL), *n* (%)				0.338
≤20 ng/mL	50 (43.9)	13 (37.1)	37 (46.8)	
>20 ng/mL	64 (56.1)	22(62.9)	42 (53.2)	
CA19-9 (U/)mL, *n* (%)				**0.0105**
≤37 U/mL	69 (60.5)	15 (42.9)	54 (68.3)	
>37 U/mL	45 (39.5)	20 (57.1)	25 (31.7)	
CEA (ng/mL), *n* (%)				0.124
≤3 ng/mL	56 (49.1)	21 (60.0)	35 (44.3)	
>3 ng/mL	58 (50.9)	14 (40.0)	44 (55.7)	
ALT (U/l), *n* (%)				0.995
≤33 U/l	13 (11.4)	4 (11.4)	9 (11.4)	
>33 U/l	101 (88.6)	31 (88.6)	70 (88.6)	
AST (U/l), *n* (%)				0.527
≤32 U/l	16 (14.0)	6 (17.4)	10 (12.7)	
>32 U/l	98 (86.0)	29 (82.9)	69 (87.3)	
GGT (U/l), *n* (%)				0.928
≤40 U/l	19 (16.7)	6 (17.1)	13 (16.5)	
>40 U/l	95 (83.3)	29 (82.9)	66 (83.5)	
Tumour size, *n* (%)				**0.048**
≤4 cm	54 (47.4)	12 (34.3)	42 (53.2)	
>4 cm	60 (52.6)	23 (65.7)	37 (46.8)	
Tumour nodules, *n* (%)				**0.045**
Single	76 (66.7)	28 (80.0)	48 (60.8)	
Multiple	38 (33.3)	7 (20.0)	31 (39.2)	
Tumour differentiation, *n* (%)				0.815
G1	1 (0.9)	0	1 (1.3)	
G2	109 (95.6)	34 (97.1)	75 (94.9)	
G3	3 (2.6)	1 (2.9)	2 (2.5)	
G4	1 (0.9)	0	1 (1.3)	
Child pugh, *n*(%)				0.593
A	91 (79.8)	29 (82.9)	62 (78.5)	
B	23 (20.2)	6 (17.1)	17 (21.5)	

* Mante–Haenszel corrected, 2-tailed P.

## Data Availability

The original contributions presented in the study are included in the article. Further inquiries can be directed to the corresponding author.

## Supplementary Materials

The following are available online at https://www.mdpi.com/article/10.3390/cancers13153771/s1, Figure S1: Limit of detection (LOD) for TERTp −124A detection; Figure S2: Assessment of linear range and precision of ddPCR for low target concentration; Figure S3: False-positive evaluation and limit of blank (LOB); Table S1: dMIQE2020 checklist for authors, reviewers, and editors.
