# Supplementary material for: Clinical Significance of Telomerase Reverse-Transcriptase Promoter Mutations in Hepatocellular Carcinoma
Source: Cancers (Basel). 2021 Jul 27;13(15):3771. doi: 10.3390/cancers13153771 (PMC8345216; doi:10.3390/cancers13153771)
Supplement: Supplementary file 1 [file cancers-13-03771-s001.zip › cancers-1281139-supplementary.pdf]

Table S1. dMIQE2020 checklist for authors, reviewers and editors.

ITEM TO CHECK	PROVIDED	COMMENT
	**Y/N**	
**1. SPECIMEN**		
Detailed description of specimen type and numbers	Y	Liver biopsies of HCV-related HCC (n= 99), HBV-related HCC (n=10), non-virus related HCC (n=5), HCV-related CC (n=5), HCC-CC (n=2), peri-tumor cirrhotic liver tissues (n=23), normal liver tissues (n=11).
Sampling procedure (including time to storage)	Y	At the time of resection each liver biopsy was divided by a pathologist in two sections: the first section was immediately submerged in RNAlater® solution (Ambion, Inc.) while the second was subjected to histopathologic examination
Sample aliquotation, storage conditions and duration	Y	Samples were stored at -80°C until analysis
**2. NUCLEIC ACID EXTRACTION**		
Description of extraction method including amount of sample processed	Y	Approximately 60mg of liver tissue is digested with proteinase K (150 µg/ml) in 500 µl of lysis buffer (10 mM Tris-HCL, pH 7.6, 5 mM EDTA, 150 mM NaCl, 1% SDS) at 37°C, over-night. DNA is purified with phenol-chloroform-isoamyl alcohol (25:24:1) and precipitated with 0.3 M sodium acetate (pH 4.6) in ethanol.
Volume of solvent used to elute/resuspend extract	Y	200-300 µL TE
Number of extraction replicates	Y	1-2
Extraction blanks included?	Y	Extraction of blanks are included every 5 samples
**3. NUCLEIC ACID ASSESSMENT AND STORAGE**		
Method to evaluate quality of nucleic acids	Y	See below
Method to evaluate quantity of nucleic acids (including molecular weight and calculations when using mass)	Y	Quantity of DNA samples are determined by Nanodrop 2000c spectrophotometer (Thermo Fisher Scientific, Waltham, Massachusetts). Quality is assessed by the ratio of absorbance at 260 nm and 280 nm required to be above 1.8
Storage conditions: temperature, concentration, duration, buffer, aliquots	Y	3-5 aliquots of each DNA sample (200-500ng/µL TE) are stored at -80°C for two years
Clear description of dilution steps used to prepare working DNA solution	Y	Working DNA solution (50ng/µL) is obtained by calculating the dilution factor ([DNA]ng/50ng) and by adding the stock DNA to the required TE volume.
**4. NUCLEIC ACID MODIFICATION**		

Template modification (digestion, sonication, pre-amplification, bisulphite etc.)	N	
Details of repurification following modification if performed	N	
**5. REVERSE TRANSCRIPTION**	**N**	
cDNA priming method and concentration		
One or two step protocol (include reaction details for two step)	N	
Amount of RNA added per reaction	N	
Detailed reaction components and conditions	N	
Estimated copies measured with and without addition of RT*	N	
Manufacturer of reagents used with catalogue and lot numbers	N	
Storage of cDNA: temperature, concentration, duration, buffer and aliquots	N	
**6. dPCR OLIGONUCLEOTIDES DESIGN AND TARGET INFORMATION**		
Sequence accession number or official gene symbol	Y	Accession number AH007699.2
Method (software) used for design and *in silico* verification	Y	Beacon Designer Software (Premier Biosoft International, Palo Alto, CA)
Location of amplicon	Y	nt 11131 - 11186
Amplicon length	Y	66bp
Primer and probe sequences (or amplicon context sequence)**	Y	TERT F 5’-CGCGGAAAGGAAGGG<br>TERT R 5’-ACCCCTCCCGGGTCC<br>TERT mut 5’-CCCGGAAGGGGCTGGG<br>TERT WT 5’-CCCGGAGGGGGCTGG
Location and identity of any modifications	N	
Manufacturer of oligonucleotides	Y	Bio-Rad Laboratories, USA
**7. dPCR PROTOCOL**		
Manufacturer of dPCR instrument and instrument model	Y	QX200 Digital droplet PCR System, Bio-Rad Laboratories
Buffer/kit manufacturer with catalogue and lot number	Y	ddPCR Supermix for Probes (No dUTP) #1863024
Primer and probe concentration	Y	Primer=18µM<br>Probe=5 µM
Pre-reaction volume and composition (incl. amount of template and if restriction enzyme added)	Y	20µl final volume comprising:<br>10µl (2x) ddPCR Super Mix<br>1µl (20x) wild type probe/primers mix,<br>1µl (20x) mutant probe/primers mix,<br>2µl of 5 M Betaine (Sigma Aldrich)<br>100ng DNA<br>deionized distilled water to 20 µl.<br>No addition of restriction endonuclease
Template treatment (initial heating or chemical denaturation)	N	
Polymerase identity and concentration, Mg++ and dNTP concentrations***	N	
Complete thermocycling parameters	Y	1. Denaturation: 10 min 95˚C;<br>2. 40 cycles of 1 min annealing/ extension at 55 ˚C and 30 s denaturation at 94 ˚C;

		3. Denaturation 10 min at 98 ˚C<br>4. Holding at 4 ˚C.<br>5. Rate of temperature rise 2 ˚C/s.
**8. ASSAY VALIDATION**		
Details of optimisation performed	Y	Temperature gradient (52-60 °C)<br>Betaine concentration (1M-0.1M)
Analytical specificity (vs. related sequences) and limit of blank (LOB)	Y	Figure S1, Figure S2, Figure S3
Analytical sensitivity/LoD and how this was evaluated	Y	Figure S1, Figure S2, Figure S3
Testing for inhibitors (from biological matrix/extraction)	N	
**9. DATA ANALYSIS**		
Description of dPCR experimental design	Y	See material and Methods
Comprehensive details negative and positive of controls (whether applied for QC or for estimation of error)	Y	Figure S1, Figure S2, Figure S3
Partition classification method (thresholding)	N	Manual
Examples of positive and negative experimental results (including fluorescence plots in supplemental material)	Y	Figure 1, Figure S1, Figure S2, Figure S3
Description of technical replication	Y	Figure 1, Figure S1, Figure S2, Figure S3
Repeatability (intra-experiment variation)	Y	Figure 1, Figure S1, Figure S2, Figure S3
Reproducibility (inter-experiment/user/lab etc. variation)	Y	Figure 1, Figure S1, Figure S2, Figure S3
Number of partitions measured (average and standard deviation)	Y	14,094 (± 1426)
Partition volume	N	
Copies per partition (λ or equivalent) (average and standard deviation)	N	
dPCR analysis program (source, version)	Y	See Material and Methods
Description of normalisation method	N	
Statistical methods used for analysis	Y	See Material and Methods
Data transparency	raw data available on request	m.tornesello@istitutotumori.na.it

* Assessing the absence of DNA using a no RT assay (or where RT has been inactivated) is essential when first extracting RNA. Once the sample has been validated as DNA-free, inclusion of a no-RT control is desirable, but no longer essential.

** Disclosure of the primer and probe sequence is highly desirable and strongly encouraged. However, since not all commercial pre-designed assay vendors provide this information when it is not available assay context sequences must be submitted (Bustin et al. Primer sequence disclosure: A clarification of the MIQe guidelines. Clin Chem 2011;57:919-21.)

*** Details of reaction components is highly desirable, however not always possible for commercial disclosure reasons. Inclusion of catalogue number is essential where component reagent details are not available.

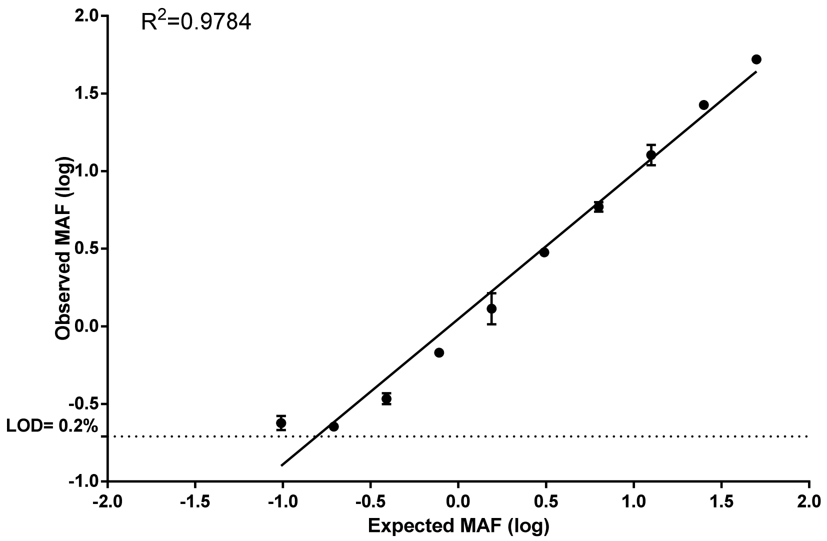

Figure S1. Limit of detection (LOD) for TERTp -124A detection. Observed versus expected MAFs in mutant serial dilutions (50%; 25%; 12.5%; 6.25%; 3.12%; 1.56%; 0.78%; 0.39%; 0.19%; 0.095%). Dashed line: limit of detection (LOD) = 0.2%.

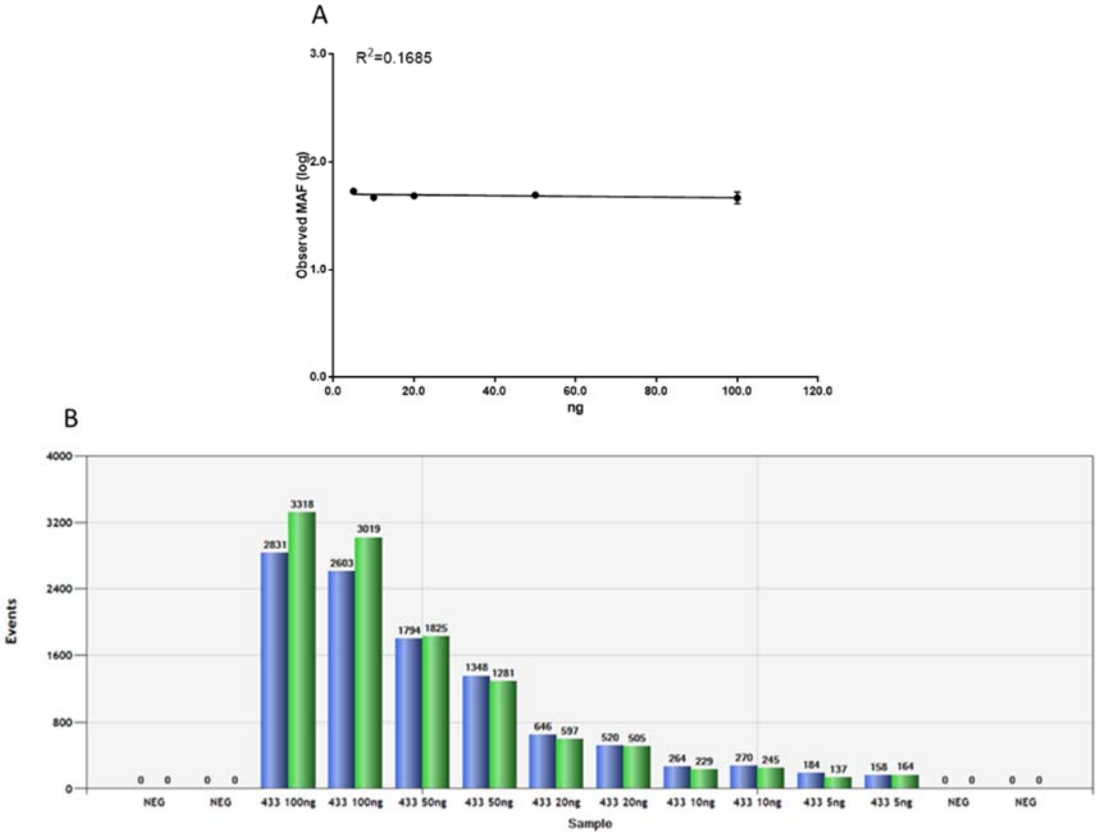

Figure S2. Assessment of linear range and precision of ddPCR for low target concentration. A) Observed MAFs in mutant serial dilutions (1:2) from 100 ng down to 5 ng of sample 433. B) Number of TERTp WT (blue) and TERTp -124° (green) in each reaction.

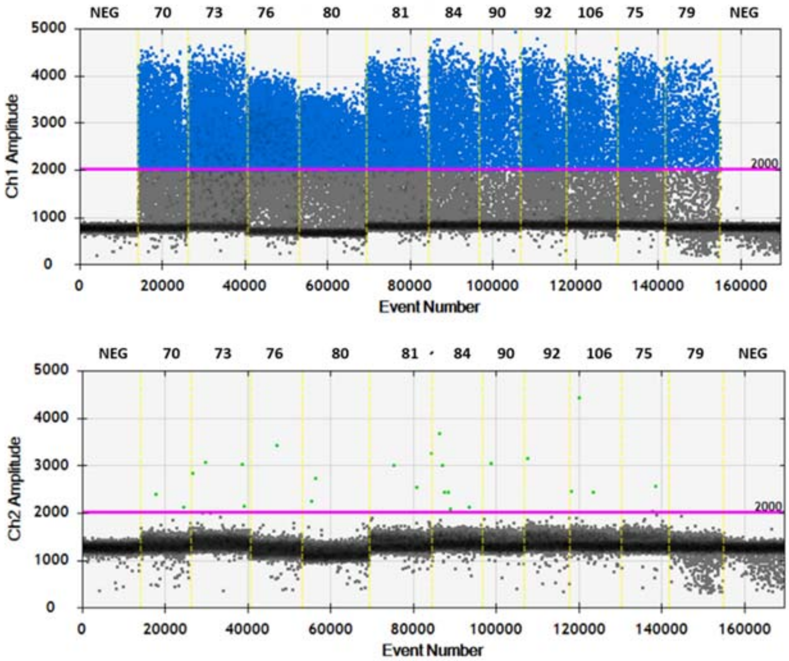

Figure S3. False-positive evaluation and limit of blank (LOB) in genomic DNA extracted from 11 liver tissues from healthy donors (negative controls). The DNA target was 100ng for each sample. The mean number of false positive events was 2.2 (±1.7).
